# Discriminating ecological processes affecting different dimensions of α‐ and β‐diversity in desert plant communities

**DOI:** 10.1002/ece3.8710

**Published:** 2022-03-18

**Authors:** Lamei Jiang, Dong Hu, Hengfang Wang, Guanghui Lv

**Affiliations:** ^1^ 47907 College of Resources and Environmental Science Xinjiang University Urumqi China

**Keywords:** alpha diversity, beta diversity, ecological restoration, nestedness, pattern, turnover

## Abstract

Understanding the spatial distribution of plant diversity and its drivers are major challenges in biogeography and conservation biology. Integrating multiple facets of biodiversity (e.g., taxonomic, phylogenetic, and functional biodiversity) may advance our understanding on how community assembly processes drive the distribution of biodiversity. In this study, plant communities in 60 sampling plots in desert ecosystems were investigated. The effects of local environment and spatial factors on the species, functional, and phylogenetic α‐ and β‐diversity (including turnover and nestedness components) of desert plant communities were investigated. The results showed that functional and phylogenetic α‐diversity were negatively correlated with species richness, and were significantly positively correlated with each other. Environmental filtering mainly influenced species richness and Rao quadratic entropy; phylogenetic α‐diversity was mainly influenced by dispersal limitation. Species and phylogenetic β‐diversity were mainly consisted of turnover component. The functional β‐diversity and its turnover component were mainly influenced by environmental factors, while dispersal limitation dominantly effected species and phylogenetic β‐diversity and their turnover component of species and phylogenetic β‐diversity. Soil organic carbon and soil pH significantly influenced different dimensions of α‐diversity, and soil moisture, salinity, organic carbon, and total nitrogen significantly influenced different dimensions of α‐ and β‐diversity and their components. Overall, it appeared that the relative influence of environmental and spatial factors on taxonomic, functional, and phylogenetic diversity differed at the α and β scales. Quantifying α‐ and β‐diversity at different biodiversity dimensions can help researchers to more accurately assess patterns of diversity and community assembly.

## Graphical abstract

Environmental filtering mainly influenced species richness and Rao quadratic entropy; phylogenetic α‐diversity was mainly influenced by spatial factors. Species and phylogenetic β‐diversity were mainly influenced by turnover. Functional β‐diversity and its turnover component are largely determined by niche process. Species and phylogenetic diversity are largely determined by spatial factors. Soil organic carbon and soil pH significantly influenced different dimensions of α‐diversity Soil moisture, salinity, organic carbon, and total nitrogen are significant predictors of different aspects and components of β‐diversity.

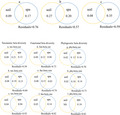

## 1 INTRODUCTION

Biodiversity patterns and the mechanisms involved in their formation are central to the study of community ecology (Anderson et al., 2011); understanding how these mechanisms are formed, maintained, and lost can contribute to the sustainable and effective conservation of biodiversity. In addition to species and functional diversity (Ricotta, 2005), phylogenetic diversity (Webb et al., 2002) is also an important component of biodiversity (de Bello et al., 2010). Distribution patterns of biodiversity are often discerned based on species richness (Hill et al., 2018; Ricklefs, 2004). However, studies have found that species‐based approaches do not take into account the fact that biomes are composed of taxa with different evolutionary histories and functional roles in ecosystems (Cardoso et al., 2014), which leads to a biased understanding of community assembly processes (Heino & Tolonen, 2017a). Mismatches and consistency between functional, phylogenetic, and species diversity have been documented in biomes (Devictor et al., 2010). In contrast to functional diversity, although species diversity, is easy to measure, it may be an incomplete indicator for the use of ecological niche space. This occurs because taxa may sometimes have similar functional roles at different sites (Villéger et al., 2012). A mismatch between functional and phylogenetic diversity also occurs from time to time, possibly because not all traits have a significant phylogenetic signal. Functional diversity calculated from small numbers of traits can appear weakly related to phylogenetic diversity (Cadotte et al., 2017; Tucker et al., 2018), however, Cadotte et al. (2019) found that finding a significant positive correlation between phylogenetic and functional diversity. The explanation is either that, most traits have phylogenetic signal, or that the presence of outlier species (those that are evolutionarily and functionally very distinct) drives this correlation. Integrated studies of species, functional, and phylogenetic diversity can help researchers to better understand the patterns of change in plant diversity (Butterfield et al., 2013; Spasojevic et al., 2014) and provide a scientific basis for the conservation of plant diversity.

Biodiversity can be characterized by dividing regional diversity into α‐ and β‐diversity (Jost, 2007; Ricotta & Szeidl, 2009). The α‐diversity represents the number and evenness of distribution of species, the value and range of species or organismal traits, and species affinities within a community (Mason & de Bello, 2013; Rosauer et al., 2009). Meanwhile, β‐diversity refers to the variability among communities at spatial and temporal scales in terms of species composition, evolutionary relationships, and functional attributes, including species, functional, and phylogenetic β‐diversity (Qin et al., 2019). Total species β‐diversity can be further divided into species turnover and nesting components (Baselga, 2010). Species turnover refers to the turnover of species from one site to another, resulting in a smaller proportion of co‐occurring species in both communities (Baselga, 2012; Bergamin et al., 2017), whereas the nested component of species β‐diversity refers to the difference in species richness between two communities, where the less abundant community is a richer subset of the richer community (James et al., 2012). Similarly, functional and phylogenetic β‐diversity can be decomposed into functional turnover and functional nestedness versus phylogenetic turnover and phylogenetic nestedness. Thus, β‐diversity may capture the dynamic nature of biodiversity patterns better than simple measures of α‐diversity alone (Socolar et al., 2016).

The majority of theories related to explaining diversity gradients can largely be summarized into two classes: niche and neutral theories. Niche theory emphasizes the importance of the contemporary environment, such as abiotic (e.g., soil attributes) and biotic factors (Chase & Leibold, 2003; Ulrich et al., 2014). Neutral theory holds that all individuals with the same trophic level in a community are equivalent in niche, that community dynamics is a random process, and it is considered that dispersal limitations play a role in the community structure (Hubbell, 2001). Thus, the spatial distance between sites has been commonly used to evaluate dispersal limitations (Liu et al., 2015; Tang et al., 2012). For example, stochastic dispersal may have a strong influence on α‐diversity, while environmental filtering and historical processes may strongly influence β‐diversity (Cavender‐Bares et al., 2009). Dispersal can also affect β‐diversity; environmental filtering and historical processes can also affect α‐diversity. They are all connected in community assembly processes (HilleRisLambers et al., 2012). Another study found that taxonomic, functional, and phylogenetic diversity at the α scale were determined by ecological niche processes, while phylogenetic diversity at the β scale was related to spatial factors (Wang et al., 2019). Dispersal limitation mainly influenced taxonomic and functional β‐diversity, while environmental filtering dominantly influenced phylogenetic β‐diversity in the Inner Mongolia grassland (Li et al., 2021). Therefore, analyses comparing patterns of different aspects of diversity at different scales may be necessary to identify ecological processes.

In recent years, global warming has led to an increase in the frequency of extreme weather events in arid regions and the expansion of arid zones (Dai, 2013), and droughts are expected to intensify in the future. This may lead to changes in the structure and function of desert ecosystems, affecting ecosystem services and human well‐being (Oliveira et al., 2020). Xinjiang is located in the northwestern borderlands of China and has a large geographical area with arid and semi‐arid zones. Despite the richness of vegetation types throughout Xinjiang, the ecological environment is generally fragile and extremely sensitive to the effects of climate change and human activities. The Ebinur Lake Basin Wetland National Nature Reserve is a treasure trove of biodiversity in the arid desert region of Xinjiang and provides a good site for the study of biodiversity. The patterns and causes of diversity changes in plant communities in this desert ecosystem have been well documented, such as differences in the habitat preferences of different species groups, and the significant positive effects of soil total nitrogen and sulfur content on plant diversity (Zhang et al., 2018). Drought significantly affects functional α‐diversity, and the effects of drought on the phylogenetic structure of desert plants are greater than those of salinity (Gong et al., 2019). Environmental filtration and dispersal limitation explain β‐diversity on different scales, dispersal limitation has stronger effects on species β‐diversity on small and medium sampling scales, while environmental filtration plays a dominant role on a large scale; for the different components, environmental filtering better explained the total β‐diversity and species turnover, while dispersal limitation had a greater effect on the species nestedness (Hu et al., 2021). However, previous studies have focused on species, functional, and phylogenetic α‐diversity and have only discussed their relationship with environmental factors, with less of a distinction made between the effects of neutral processes on these types of α‐diversity during ecology, and fewer studies on β‐diversity. Previous analyses of the drivers of β‐diversity pattern formation have focused only on β‐diversity itself, without distinguishing between the turnover and nesting components that shape β‐diversity.

The distribution characteristics and patterns of change in plants in the Ebinur Lake Basin Wetland National Nature Reserve are influenced by environmental factors, especially soil moisture content (Gong et al., 2019; Zhang et al., 2018), which can provide basic theoretical support for studying changes in diversity. However, diversity studies based on multiple dimensions can provide a more accurate and complete assessment of compositional changes and identify the most suitable locations for biodiversity conservation. With this in mind, to compare the drivers of species, functional, and phylogenetic diversity at the α and β scales, we placed 60 sample squares perpendicular to the Aqikesu River. Correlations between α‐diversity were calculated and analyzed from measurements, and the role of environmental filtering and dispersal limitation on α‐diversity in different dimensions was explored. We then disentangled the β‐diversity to explore the ecological processes affecting the different dimensions of β‐diversity and its components. Specifically, we sought to address three specific questions: (1) What are the relative importance of environmental filters and dispersal limitation to α‐diversity? (2) What are the patterns of variation in the components of species, functional, and phylogenetic β‐diversity? (3) Which of the environmental filters and dispersal limitation play a dominant role in β‐diversity? By exploring these questions, it is hoped that a fuller picture of the underlying mechanisms that shape patterns of variation in species composition, functional traits, and phylogenetic relationships within and between arid zone communities can be revealed.

## 2 MATERIALS AND METHODS

### 2.1 Overview of the research area

The Ebinur Lake National Wetland Nature Reserve is located in northwestern Jinghe County, Xinjiang Uygur Autonomous Region, China (82°36′–83°50′ E, 44°30′–45°09′ N). The climate is dry in the reserve, with an average annual precipitation of about 100  mm, evaporation of over 1600  mm, and extreme maximum, extreme minimum, and average annual temperatures of 44°C, −33°C, and 6–8°C (Yang et al., 2014), which is typical of a temperate continental arid climate (Gong et al., 2017). The vegetation is dominated by arid and super‐arid desert plants, with a variety of saline, sandy, and aquatic vegetation communities. The plant life type is dominated by small trees and shrubs and semi‐shrubs, and the herbaceous plants are dominated by perennial herbs, with a few short‐lived plants distributed in the desert area. The main plants in the study area are *Populus euphratica*, *Haloxylon ammodendron*, *Halimodendron halodendron*, *Reaumuria soongorica*, *Nitraria roborowskii*, *Suaeda microphylla*, *Apocynum venetum*, *Karelinia caspia*, *Alhagi sparsifolia, and Phragmites australis*.

### 2.2 Field survey and data generation

**FIGURE 1:**
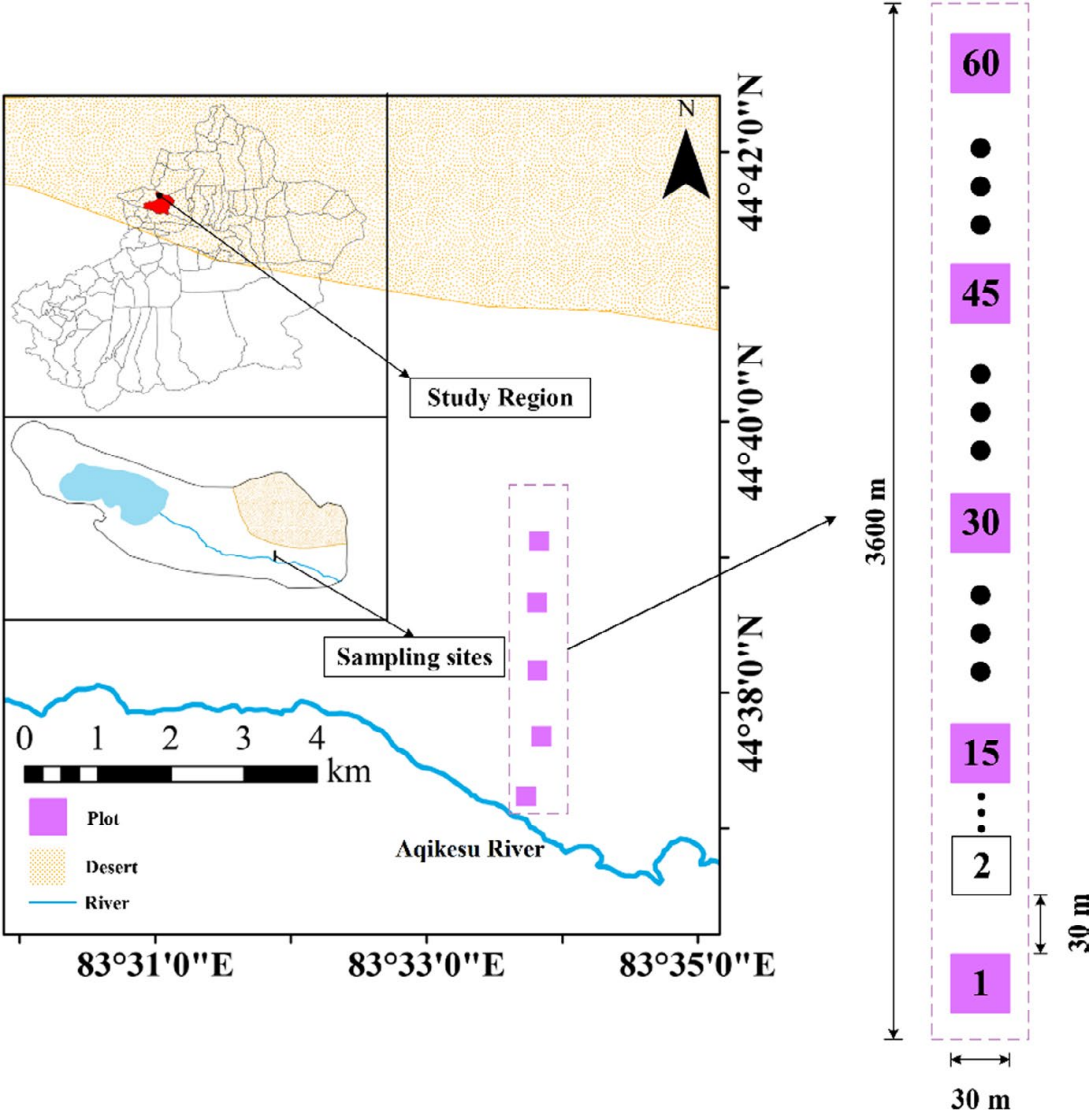
The study area and location of the plots

A 30  m  ×  3600  m belt transect was established perpendicular to the Aqikesu River, in which 60 plots measuring 30  m  ×  30  m were divided. Each plot was separated by about 30  m. Three 1  m ×1  m herbaceous quadrats were set up in each 30  m  ×  30  m plot (Figure 1). The survey consisted mainly of recording the geographical coordinates and elevation of each large sample plot, and investigating and summarizing the species and species diversity of all species occurring within the plot. Three soil sampling points were selected using a diagonal sampling method in each sample square in the study area; soil samples were collected in three soil profiles (0–10  cm, 10–20  cm, and 20–30  cm). A total of 540  soil samples were obtained. The method used for soil index determination (Bao, 2000) is shown in Table S1. The plant distribution pattern along the transect (Figure S1). The salt‐tolerant shrubs or herbaceous plants (such as *Halimodendron halodendron Suaeda microphylla*, *Suaeda salsa* and *Glycyrrhiza uralensis*) were evenly distributed near the river, only a small number of drought‐tolerant herbs or shrubs were distributed away from the river, that is, *Aeluropus pungens*, *Calligonum mongolicum*, and *Agriophyllum squarrosum*. Species richness fluctuates with along spatial distance (Figure S2).

Sites were selected in each plot where there was no mutual shade between plants, at these sites, healthy leaves from the four cardinal directions of individual plants were selected for sampling. leaves of each species were selected and their length, width, and thickness were measured using vernier calipers; 30–50 leaves of each species were collected and laid out on grid paper, photographed, and saved, and then the leaves were processed using Photoshop software to obtain their leaf area. The fresh weight was then weighed on a one‐in‐ten‐thousand balance, placed in an envelope, taken back to the laboratory, dried in an oven, and then weighed to obtain the specific leaf area and the dry matter content of the leaves.

Within each sample square, the maximum plant height of the species was measured using a ruler. About 50  g of fresh mature leaves were collected from 5 to 10 plants per species (Gong et al., 2017) and taken back to the laboratory to be dried to a constant weight at 70°C for determination of the carbon, nitrogen, and phosphorus content of the leaves. Fruit types and leaf phenotypic traits for each species were obtained by searching the Flora of China database (http://www.iplant.cn).

The Chinese nomenclature of the species surveyed in the field was checked against the Flora of China database and their Latin scientific names were recorded; the corrected plant list was collated into a “family/genus/species” format using the R language plantlist package (Zhang, 2018); this list was compiled using the Phylomatic (http://phylodiversity.net/phylomatic) tool to construct a phylogenetic tree based on the method used by Zanne et al. (2014).

### 2.3 Data analysis

#### 2.3.1 Calculation of α‐diversity

Species α‐diversity was measured using species richness, because this is the most widely used metric for measuring species α‐diversity. The Rao quadratic entropy (Rao Q), which was chosen as the functional α‐ diversity index, contains both a measure of the relative abundance of species and pairwise functional differences between species (Mason et al., 2012); it can be a good indicator of changes in functional traits in the community. The mean pairwise phylogenetic distance MPD index, which predicts the mean phylogenetic distance between all pairs of taxonomic units in a clustering tree (Webb et al., 2008), was chosen to illustrate the overall clustering of the evolutionary tree (Webb et al., 2002) Mean phylogenetic distance between species (MPD) is the most widespread metrics applied at the level of individual samples (α‐diversity) (Yakimov et al., 2020). Species, functional, and phylogenetic α‐diversity calculations were performed by the vegan, FD, and picante packages, respectively. Correlation analyses between the three types of diversity were performed in the stats packages.

#### 2.3.2 Calculation of β‐diversity and its components

The Sørensen index of dissimilarity (Tax.beta.sor) was used to measure similarity in species composition, that is, species β‐diversity (Baselga, 2010). In addition, Tax.beta.sor reflects overall differences between samples; The Simpson index of dissimilarity (Tax.beta.sim) indicates pure species turnover with no differences in richness; and the nestedness‐fraction of Sørensen dissimilarity (Tax beta.sne) indicates the overall variation due to differences in richness between different samples. These indices were calculated using Equations (1), (2), and (3), respectively,

(1) $$\text{Tax}.\text{beta}.\text{sor}\;=\;\frac{b+c}{2a+b+c}$$

(2) $$\text{Tax}.\text{beta}.\text{sim}\;=\;\frac{\text{min}\;(b,c)}{a\;\text{+ min}\;(b,c)}$$

(3) $$\text{Tax}.\text{beta}.\text{sne}\;=\;\frac{\text{max}(b,c)‐\text{min}(b,c)}{2a+b+c}\times \frac{a}{a+\text{min}(b,c)}$$

where *a* is the number of species common to both samples, *b* is the number of species unique to the first sample, and *c* is the number of species unique to the second sample. The calculation of species β‐diversity and its components was performed by the beta.pair function in the betapart package.

A multidimensional functional space was then constructed by performing principal coordinate (PCoA) analysis of 11 functional traits representing plant ecological strategies using the Euclidean distance matrix. The resulting PCoA scores were used as new traits to calculate the functional β‐diversity and its components (Laliberté & Legendre, 2010; Villéger et al., 2013). When the functional space is more than four dimensions, calculating the functional β‐diversity and its components takes long time, and the function requires that the species richness of each sample is strictly greater than the functional dimension. Therefore, the dimensions of the functional space were limited to the first four PCoA axes in this study (Gómez‐Rodríguez & Baselga, 2018). The above analyses were performed in the ape, vegan, and betapart packages of the R language. The results of the functional trait principal coordinates analysis are presented in Table S2.

Phylogenetic β‐diversity can be calculated analogously to species diversity (Leão‐Pires et al., 2018). In this study, phylogenetic β‐diversity (phy.beta.sor) was decomposed into phylogenetic turnover (phy.beta.sim) and phylogenetic nestedness (phy.beta.sne) components. The calculation of the phylogenetic β‐diversity and its components was performed by the phylo.beta.pair function in the betapart package.

#### 2.3.3 Acquisition of spatial factors

To obtain spatial factors (related to dispersal limitation), we used the adespatial package in R for Moran’s Eigenvector Maps (MEMs) analysis. Based on latitude and longitude coordinates, spatial vectors (MEMs) were created to quantify the spatial pattern of the sample, and only MEMs with a positive autocorrelation were retained as spatial predictors in the constrained ranking analysis (Borcard et al., 2011). The MEMs with large eigenvalues indicate broad‐scale spatial patterns and low eigenvalues represent fine‐scale spatial patterns in metacommunity structure (Borcard et al., 2004).

#### 2.3.4 Moran spectral randomization‐Mantel test

The Moran spectral randomization (MSR)‐Mantel test was used to explore species, functional, and phylogenetic β‐diversity and the correlations between their respective components. The Mantel test was performed using the ade4 and vegan packages, and the MSR was generated by the adespatial package in R.

#### 2.3.5 Redundancy analysis and variance partitioning

Distance‐based redundancy analysis (db‐RDA) was used to investigate the influence of β‐diversity and its components, and variance partitioning was used to analyze the relative importance of environmental and spatial factors.

First, the matrixes of β‐diversity, turnover components, and nested components were constrained and ranked using PCoA to obtain the eigenvectors; then the PCoA eigenvectors were used as response variables and the environmental factors and spatial eigenvectors (MEMs) were used as explanatory variables for RDA. Finally, the relative importance of the environmental and spatial factors was explored using variance partitioning analysis. Prior to variable screening, the significance of the full RDA model was tested using the “anova” function in the vegan package; only when the full model was significant did the “ordistep” function in the vegan package continue to be used for pre‐selection. The “ordistep” function in the vegan package was used to select the environmental and spatial factors that significantly affected each of the three types of β‐diversity and their components. The variance partitioning analysis was performed using the “varpart” function and principal coordinate analysis was conducted using the “PCoA” function of the ape package in R. The adjusted *R* ^2^ was used in the study because it is more accurate (Peres‐Neto et al., 2006).

All statistical analyses and were performed in the R software v. 4.0.3 (R Development Core Team).

## 3 RESULTS AND ANALYSIS

### 3.1 Plant α‐diversity in desert ecosystems

**FIGURE 2:**
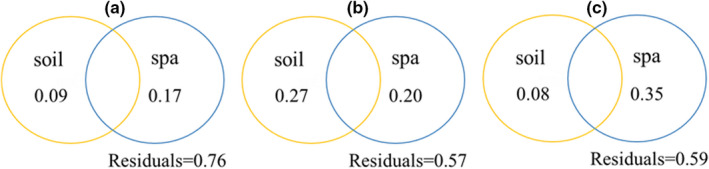
The effect of variance partitioning environmental and spatial factors on α‐diversity Note: Soil represents soil environmental factor; Spa represents spatial factor (MEMs); pH and SOC represent soil pH and soil organic carbon, respectively. a, b, and c note species richness, Rao quadratic entropy, and mean pairwise phylogenetic distance

The results of the variance partitioning reflect the relative contributions of environmental and spatial factors to species, functional, and phylogenetic α‐diversity (Figure 2). Environmental variables alone explained more of the variance in species richness and Rao quadratic entropyRao quadratic entropy (19% and 27%, respectively) than spatial variables alone (7% and 20%, respectively); phylogenetic α‐diversity was explained by spatial variables alone (35%) to a much greater extent than the effect of environmental variables alone (8%). The environmental factor that significantly influenced species richness was soil organic carbon, with spatial variables MEM11 and MEM18; soil organic carbon was the main environmental factor influencing Rao quadratic entropy, with spatial variables MEM13, MEM18, and MEM23. The environmental factor that significantly influenced phylogenetic α‐diversity was soil pH, with spatial variables MEM1, MEM7, MEM9, MEM13, and MEM18.

**FIGURE 3:**
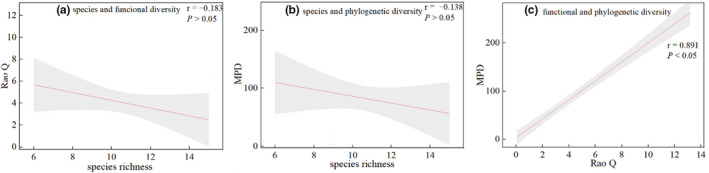
The relationships among species, functional, and phylogenetic α‐diversity

The functional and phylogenetic α‐diversity were negatively correlated with species richness, the correlation coefficients were −0.183 and −0.138, functional and phylogenetic α‐diversity were significantly positively correlated (Figure 3).

### 3.2 β‐diversity of desert plants

#### 3.2.1 Patterns of species, functional, and phylogenetic β‐diversity and their components in desert plants

Species β‐diversity (tax.beta.sor, mean = 0.40) was mainly contributed to by the species turnover component (tax.beta.sim, mean = 0.33), accounting for 82.5% of the total. In contrast, the species nested component (tax.beta.sne, mean = 0.07) accounted for 17.5% of β‐diversity. Functional β‐diversity (fun.beta.sor) had a mean value of 0.48, and the contributions of the functional turnover component (fun.beta.sim, mean = 0.25) and the functional nested component (fun.beta.sne, mean = 0.23) to functional β‐diversity were not significantly different, at 52.08% and 47.92%, respectively. Phylogenetic β‐diversity (phy.beta.sor, mean = 0.20) was mainly contributed to by the phylogenetic turnover component (phy.beta.sim, mean = 0.14) with 70%, compared to the phylogenetic nested component (phy.beta.sne, mean = 0.06), which contributed to 30% of β‐diversity.

A Mantel correlation test revealed significant correlations between species, functional, and phylogenetic β‐diversity and their components in desert plants (Figure 5). The correlation coefficients between species β‐diversity (tsor) and functional (fsor) and phylogenetic β‐diversity indices (psor) were 0.608 and 0.686, respectively, while the correlation coefficient between functional (fsor) and phylogenetic β‐diversity indices (psor) was 0.429, the correlation coefficient between species and functional β‐diversity turnover components was 0.684, and the correlation coefficient between nested components was 0.452. The correlation coefficient between the functional and phylogenetic β‐diversity turnover components was 0.459 and the correlation coefficient between the nested components was 0.454. The correlation coefficient between the species diversity and phylogenetic β‐diversity turnover components was 0.632 and the correlation coefficient between the nested components was 0.598.

#### 3.2.2 Drivers of species, functional, and phylogenetic β‐diversity and their components in desert plants

**FIGURE 4:**
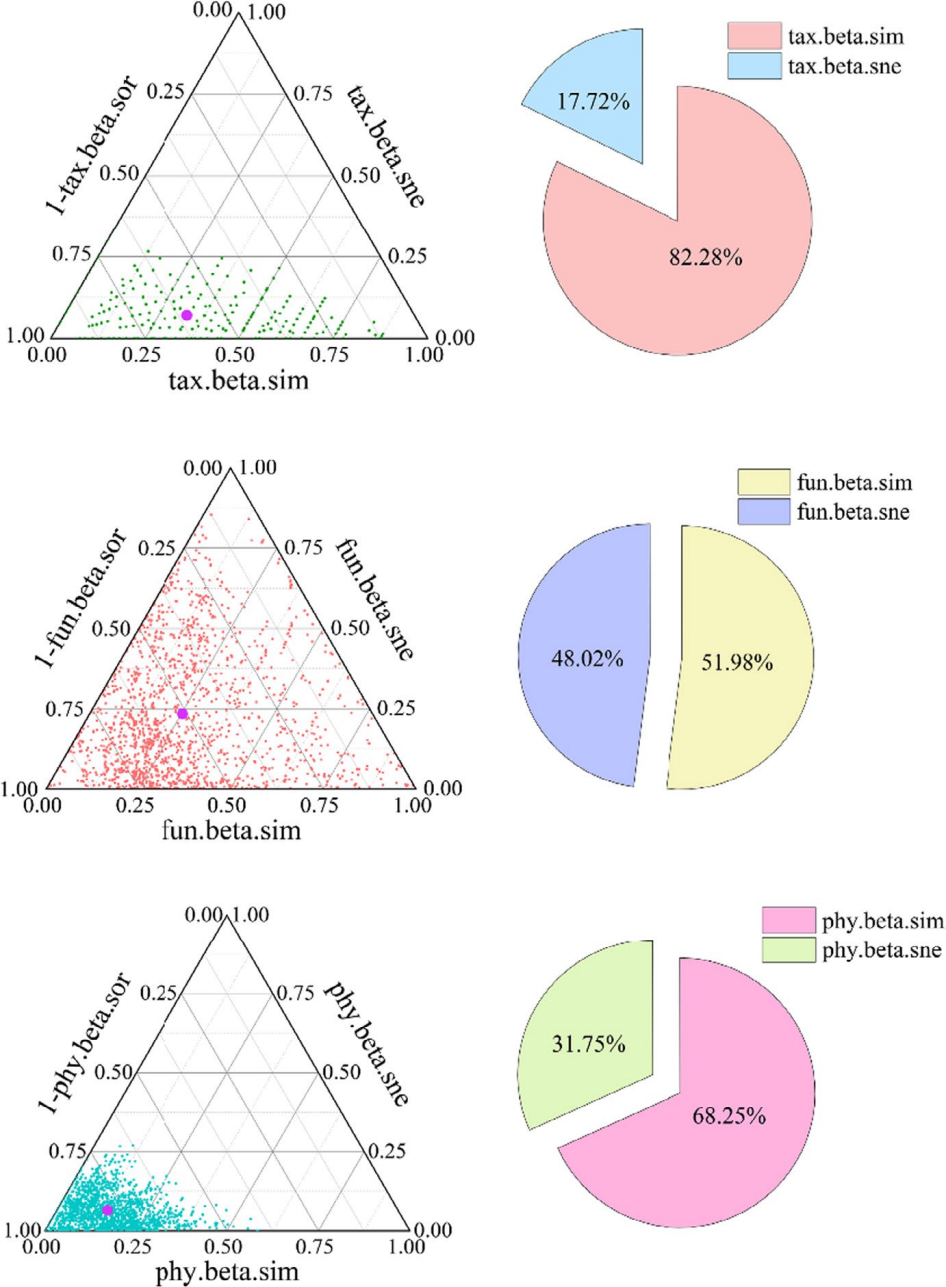
The patterns and proportion of species, function, and phylogenetic β‐diversity components of desert plants. Note: Each green, red, and blue dot represent a pair of sites in triangular plots. Their positions were determined by a triplet of values from the species, functional and phylogenetic composition similarity (1‐taxa.beta.sor, 1‐fun.beta.sor, and 1‐phy.beta.sor, respectively), tax.beta.sor: species β‐diversity, tax.beta.sim: species turnover component; tax.beta.sne: species nestedness component; fun.beta.sor: functional β‐diversity, fun.beta.sim: functional turnover component; fun.beta.sne: functional nestedness component; phy.beta.sor: phylogenetic β‐diversity, phy.beta.sim: phylogenetic turnover component; phy.beta.sne: phylogenetic nestedness component, each triplet sums to 1. The larger purple dots represent the mean values of the three components in all pair of sites

The results of the variance partitioning reflect the relative effects of environmental factors and spatial structure on species, functional, and phylogenetic β‐diversity and their turnover and nested components (Figure 4). Spatial variables alone explained more of the variance in species and phylogenetic β‐diversity (13% and 16%, respectively) than environmental variables alone (8% and 2%, respectively); the same pattern was found for the turnover component of species and phylogenetic β‐diversity. In contrast, spatial variables alone explained 4% of the nested components of species β‐diversity, as did environmental variables alone, and for phylogenetic β‐diversity, the nested components were only influenced by spatial variables. However, for functional β‐diversity, environmental variables alone explained more than spatial variables alone.

The environmental factors that significantly affected species β‐diversity were total soil nitrogen and salinity, with the spatial variables MEM1, MEM2, MEM4, MEM5, and MEM14; soil water content and total nitrogen were the main environmental factors that affected the turnover component of species β‐diversity, with the spatial variables MEM1, MEM2, MEM5, and MEM14; and the environmental factors that affected their nested components and spatial variables were soil organic carbon and MEM18. The main environmental factors affecting functional β‐diversity were soil water content and salinity, with the spatial factors MEM1, MEM2, and MEM3; the environmental factor affecting the turnover component of functional β‐diversity was soil moisture, with spatial variables MEM1 and MEM3; and the environmental factor affecting the nested component was soil salinity, with spatial factors MEM1 and MEM3. The environmental factors significantly affecting the phylogenetic β‐diversity and its turnover components were all soil water content, with the spatial variables MEM1, MEM2, MEM5, and MEM14.

## 4 DISCUSSION

Identifying the drivers that influence biodiversity in an area is particularly important for predicting ecosystem responses to environmental change (Sanitha et al., 2020). The relative importance of ecological niches and neutral processes in plant diversity has been debated (Blundo et al., 2016; Wang et al., 2019). Many studies have discussed the independent and common effects of ecological niches and neutral processes on plant diversity in different ecosystems (Li et al., 2021; Liu et al., 2015; Yakimov et al., 2020), but few studies have reported on whether the relative importance of ecological niches and neutral processes in desert ecosystems changes as diversity in different dimensions changes (Hu et al., 2021; Wang et al., 2019). Our results suggest that these diversities have inconsistent responses to the effects of ecological niches and neutral processes. These patterns promote a deeper understanding of the mechanisms that construct species, functional, and phylogenetic diversity in desert plant communities. At the α scale, environmental variables explained more of the species richness than spatial variables (Figure 2), suggesting that ecological niche processes play a major role in changes in species richness, and are mainly influenced by the soil organic carbon. There are two aspects in which soil organic carbon affects the change of plant species. On the one hand, it affects the growth of plants by affecting the release of plant nutrient elements from microorganisms (Sathya et al., 2009); on the other hand, it also affects soil physical and chemical properties such as soil particle viscosity, water‐holding capacity, aggregate structure, and air permeability, and these in turn affect the survival and growth of plants (Lai et al., 2013). Similarly, functional α‐diversity is mainly driven by soil organic carbon. As an important function of the ecosystem, high levels of soil carbon storage facilitate the acquisition of more carbon resources by leaves, resulting in higher leaf organic carbon content, increased leaf area, and increased light capture area, leading to increased photosynthetic capacity and a more rapid nitrogen uptake by plants (Pan et al., 2018). In contrast, phylogenetic α‐diversity was most strongly correlated with spatial variables (Figure 2), suggesting that the genealogy was more influenced by stochastic effects, that is, neutral processes, indicating the importance of evolutionary history, stochastic events, and ecological drift on genealogical structure. In addition, soil pH was found to have a significant effect on phylogenetic diversity. This may be a result of the fact that as soil alkalinity increases, soils tend to become hardened and less permeable, making it difficult for them to mineralize and release nutrients, while reducing the effectiveness of nutrients (Ding et al., 2019). This places plant growth under intense soil environmental stress, so species that are less salinity‐tolerant are at an increased risk of being eliminated as soil alkalinity increases, leading to changes in phylogenetic diversity (Tang et al., 2013). Species did not show significant correlations with functional and phylogenetic diversity (Figure 3), whereas phylogeny was significantly positively correlated with functional diversity. This suggests that considering species diversity alone leads to a loss of functional and phylogenetic information, which is consistent with many studies (Heino & Tolonen, 2017a).

**FIGURE 5:**
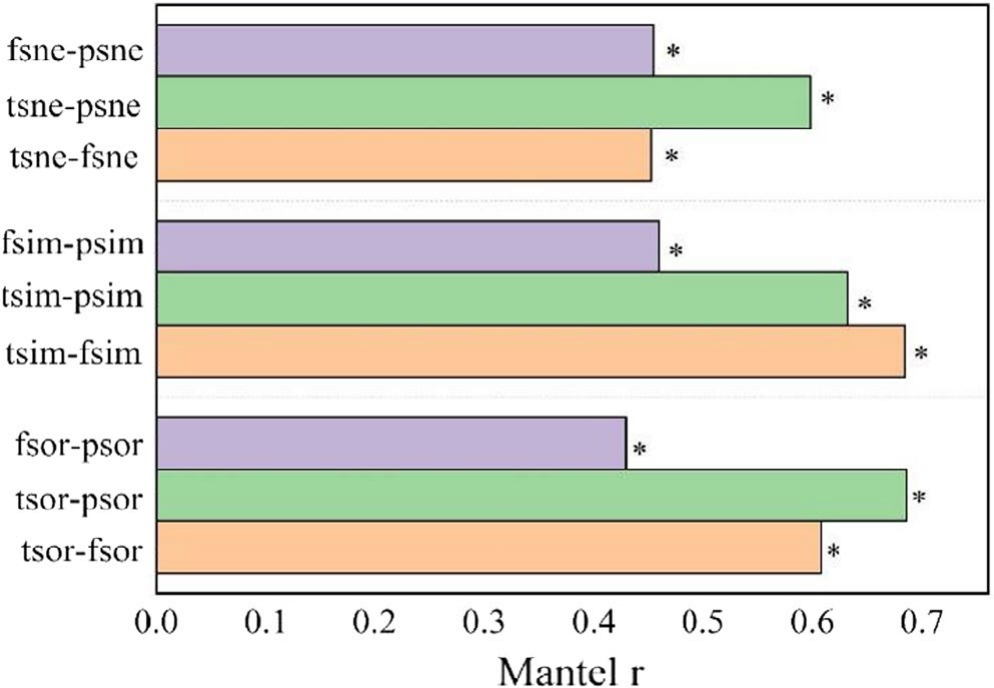
The correlation of taxonomic, functional, and phylogenetic β‐diversity components. Note: tsor: species β‐diversity, tsim: species turnover component; tsne: species nestedness component; fsor: functional β‐diversity, fsim: functional turnover component; fsne: functional nestedness component; psor: phylogenetic β‐diversity, psim: phylogenetic turnover component; psne: phylogenetic nestedness component. *p < .05

Complex interactions between different factors and processes, including historical and biogeographic constraints, environmental filtering, biotic interactions, and dispersal, affect the distribution of species in space and time (Leibold et al., 2004; McGill, 2010; Pinheiro et al., 2017), leading to changes in community composition from one location to another. Plant interactions play a crucial role in the structuring plant communities and maintaining biodiversity (Armas & Pugnaire, 2005). The facilitation increases community richness and Shannon diversity by alleviating stressful conditions in alpine meadows (Cavieres et al., 2014, 2016). Facilitators might increase trait dispersion in the local community, which could alleviate the effect of environmental filters on trait values in harsh environments, thereby contributing to ecosystem functioning (Wang et al., 2021). Interspecific competition and phylogenetic structure had weak effects on functional diversity in local dryland vegetation (Gong et al., 2019). Decomposing the contribution of turnover and nestedness to overall β‐diversity helps to overcome this complexity and can provide further insight into the community‐building mechanisms that shape total β‐diversity. For species and phylogenetic data, turnover components represent most of the total β‐diversity. Functional β‐diversity shows a less uniform pattern, where nestedness contributes less to total β‐diversity than turnover, although the contributions of the turnover and nestedness are each about 50%. Higher contributions of turnover components and lower nestedness rates have been found across many different species, habitat types, and geographic regions (Boschilia et al., 2016; Heino & Tolonen, 2017b; Soininen et al., 2018). Thus, the findings of this study may exemplify the typical pattern of additive relationships between components of β‐diversity. Soininen et al. (2018) found that spatial turnover was consistently greater than nestedness (5.7‐fold on average) from a meta‐analysis of several studies involving different biological groups, spatial and latitudinal locations, and habitat types. Traditionally, species in isolated but homogenous habitats are expected to have high levels of species β‐diversity and low levels of functional β‐diversity (Pavoine & Bonsall, 2011; Penone et al., 2016; Weinstein et al., 2014). In contrast, this study found lower species β‐diversity but higher functional β‐diversity (Figure 4), an inconsistency that may be due to some redundancy among the species studied and the different ability of species to respond to disturbances or environmental changes (Díaz & Cabido, 2001). Our study observed significant correlations between the components of species, functions, and phylogenetic β‐diversity in desert plant communities. This occurred because the composition of species and functional traits will have some phylogenetic signal. The relationship between species and phylogenetic diversity >functional and phylogenetic diversity >species and functional diversity differed in the magnitude of the three correlations (Figure 5), suggesting that these three aspects of β‐diversity were complementary. A combined approach using multiple types of diversity provided a are more comprehensive analysis than a single approach to understanding β‐diversity in desert plant community.

**FIGURE 6:**
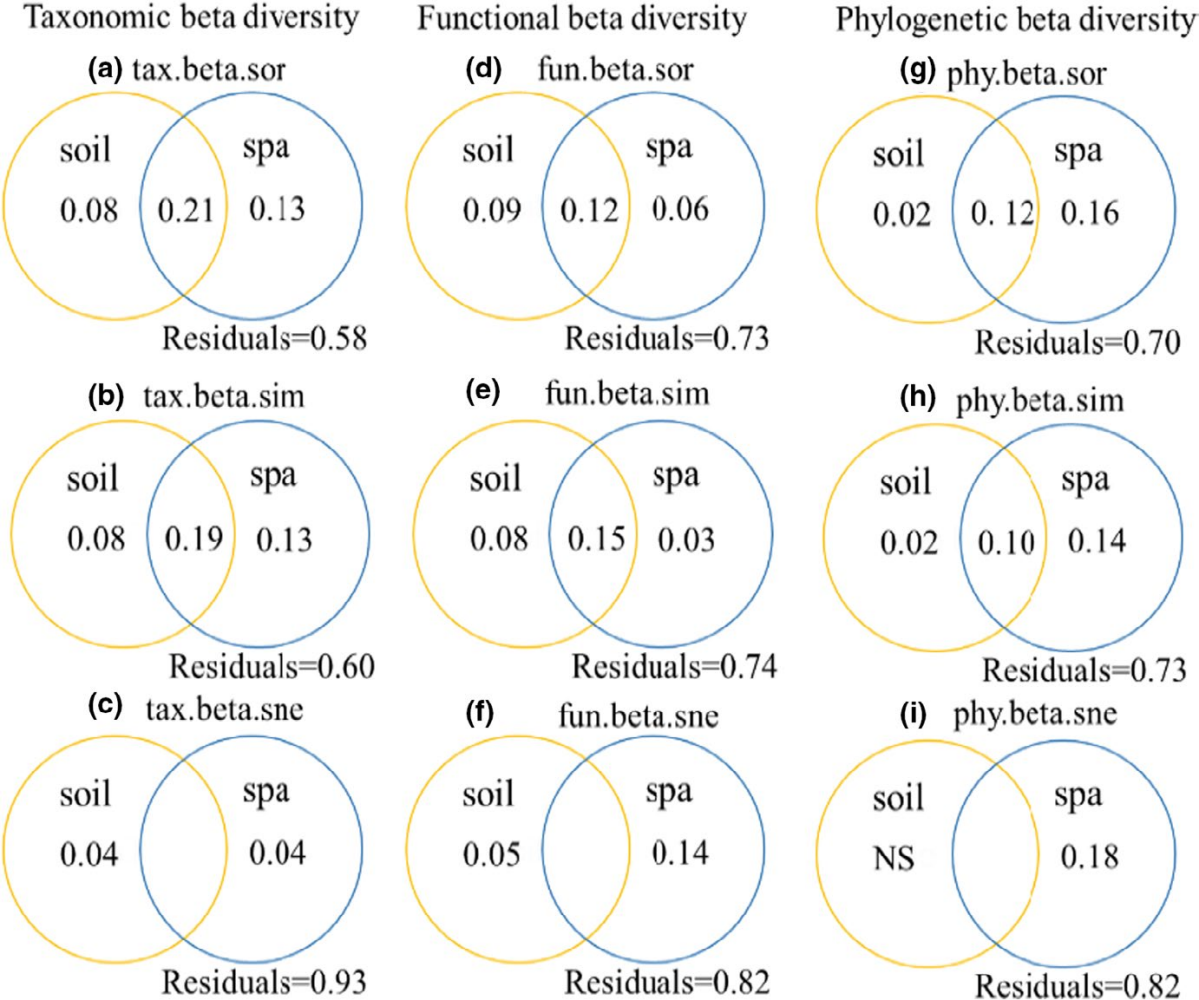
The effects of variation in partitioning environmental factors and spatial factors on β‐diversity and its components. Note: Soil represents soil environmental factor; Spa represents spatial factor (MEMs); NS represents not significant

The influence of environmental versus spatial factors on β‐diversity has been controversial over time. Some researchers have argued that environmental (such as climate, soil properties, and topography) or spatial factors are more important for β‐diversity (Jones et al., 2016; Zhao et al., 2017), while others have argued that the roles of both environmental and spatial factors are of equal importance (Qian & Shimono, 2012). Our results suggest that the relative importance of environmental and spatial variables to β‐diversity varied with the type of diversity. Spatial factors mainly influenced species and phylogenetic β‐diversity, while functional β‐diversity was mainly influenced by environmental factors, suggesting that species and phylogenetic β‐diversity were mainly influenced by neutral processes, and function was mainly influenced by ecological niche processes (Figure 6). The reason for the inconsistent performance of species and functional β‐diversity in response to environmental and spatial factors may be because different species can exhibit similar traits over long time or spatial scales (Fukami et al., 2005; Villéger et al., 2013), and traits associated with environmental tolerance and life history can alter a species’ probability of occurring in a community (Arantes et al., 2018; Holt et al., 2017). Thus, the importance of dispersal limitation and drift is expected to be higher at the species level, while the effect of environmental filtering on functional traits should be stronger (Fukami et al., 2005; Gianuca et al., 2017; Spasojevic et al., 2014). In addition, a mismatch between phylogenetic, taxonomic, and functional β‐diversity was found, caused by the fact that the impact of a species on the multidimensional β‐diversity may be a product of the interaction between its frequency of occurrence, changes in richness, the commonality or scarcity of trait values (Tobias & Monika, 2011), and the different positions of this species on the phylogenetic tree. Mismatches in phylogeny may also be caused by variations in phylogenetic signals for certain functional traits. For example, we considered traits with strong phylogenetic signals (leaf width, leaf thickness, and leaf dry matter) in addition to some unstable traits, such as specific leaf area (Wang et al., 2020). The correlation between phylogeny and functional diversity depends on the trait under consideration and the level of its phylogenetic signal (Winter et al., 2013). Therefore, when traits with different strengths of phylogenetic signals are used, there will be a risk of finding inconsistent changes in phylogenetic and functional diversity.

Species β‐diversity and its turnover components are mainly influenced by soil water content and total nitrogen content, possibly because increased nitrogen alters the availability of soil resources and changes in soil nutrients provide an advantage to species that can use additional nitrogen during interspecific competition, thus altering the species composition of the community and affecting species turnover and nestedness patterns (Xu et al., 2015). Soil moisture is a major limiting factor for plant survival in desert ecosystems, and when drought levels increase, species that can adapted to drought stress survive, while those that are less tolerant to drought are inhibited or even eliminated, leading to changes in species diversity and species turnover (Han et al., 2017). The material investment and distribution patterns of functional traits of desert plants in arid zones vary with soil water and salinity content, and plant species with different growth types have different trends and degrees of response to soil water and salinity (Qie et al., 2018), leading to changes in functional β‐diversity along the water and salinity gradients. At the same time, phylogenetic β‐diversity is mainly influenced by moisture. Drought stress can act as an environmental filter to bring closely related species together, and can also increase competition for resources between species. This results in the phylogenetic divergence of species within the community (Cavender‐Bares et al., 2009), which can lead to changes in phylogenetic diversity.

The spatial structure of the environment was more highly explained than environmental variation alone or spatial variables, suggesting that soil factors have some spatial structural properties, which are closely related to the spatial heterogeneity of soil factors in desert ecosystems (Wang et al., 2019). In addition, a proportion of the variance in the variance partitioning of the α‐ and β‐diversity and species turnover components was not explained by the known factors, and this fraction may contain some biotic and abiotic factors with nonspatial structural properties not observed in this study. The main reasons for this are (1) sampling effects due to changes in the size of local species pools (Kraft et al., 2015; Segre et al., 2014), (2) stochastic processes such as ecological drift and species extinction (Legendre et al., 2009; Segre et al., 2014), and (3) unpredictable spatial, environmental, and other types of variables (Myers et al., 2013).

As one of the repositories of biodiversity in the dry zone of Xinjiang, conducting conservation work in the Ebinur Lake Basin is important. The use of a single aspect of diversity (species richness) as a criterion for conservation should be avoided (Devictor et al., 2010). Our results show that species, functional, and phylogenetic α‐diversity are not good substitutes for each other, and therefore diversity conservation requires conservation approaches that encompass different dimensions of biodiversity. However, focusing conservation efforts on different aspects of α‐diversity alone may also be less effective. This study shows that the β‐diversity of plants consists mainly of turnover components, with a small proportion of nested components, so that conservation efforts cannot be limited to certain typical communities with high diversity indices, but large ecological reserves need to designate to cover as many community types as possible to provide for a holistic conservation approach.

## 5 CONCLUSIONS

(1) Functional and phylogenetic α‐diversity were significantly positively correlated. At the α scale, functional diversity and species richness were mainly determined by niche process, while phylogenetic α‐diversity was driven by neutral process. Soil organic carbon and soil pH were significant environmental factors that influenced different aspects of α‐diversity. (2) Functional β‐diversity and its turnover components are largely determined by niche process. Species and phylogenetic diversity are largely determined by spatial factors. Soil moisture, salinity, organic carbon, and total nitrogen are significant predictors of different aspects and components of β‐diversity. Our results suggest that management aimed at enhancing biodiversity should focus on improving and/or restoring local environmental conditions. Incorporating species, functional, and phylogenetic diversity into conservation strategies will improve their efficiency and effectiveness and maximize biodiversity conservation.

## CONFLICT OF INTEREST

The authors declare that they have no conflict of interest.

## AUTHOR CONTRIBUTIONS

**Lamei Jiang:** Investigation (equal); Methodology (equal); Resources (equal); Software (equal); Validation (equal); Visualization (lead); Writing – original draft (lead); Writing – review & editing (equal). **Dong Hu:** Investigation (equal); Methodology (supporting); Resources (equal); Software (equal). **Hengfang Wang:** Data curation (equal); Formal analysis (equal); Investigation (equal); Software (equal). **Guanghui Lv:** Funding acquisition (lead); Investigation (equal); Project administration (lead); Resources (equal); Supervision (equal).

## Supporting information

Supplementary Material

## Data Availability

Data from this study are available and can be accessed at the public data repository Dryad. https://doi.org/10.5061/dryad.v9s4mw6xz.
