# Supplementary material for: Discriminating ecological processes affecting different dimensions of α‐ and β‐diversity in desert plant communities
Source: Ecol Evol. 2022 Mar 18;12(3):e8710. doi: 10.1002/ece3.8710 (PMC8933320; doi:10.1002/ece3.8710)
Supplement: Supplementary file 1 — Supplementary Material [file ECE3-12-e8710-s001.docx]

Table S1 Soil measurement methods

| No | Indicators | Determination method |
| --- | --- | --- |
| 1 | Soil moisture content | Weight method |
| 2 | Soil salinity content | Weight method |
| 3 | pH | Potentiometry |
| 4 | Soil organic C | Potassium dichromate dilution heat method |
| 5 | Total N | Kjeldahl method |
| 6 | Ammonium N | Indophenol blue colorimetry |
| 7 | Nitrate N | Colorimetry of phenoldisulfonic acid |
| 8 | Total P | Molybdenum antimony anticolorimetric method |
| 9 | Available P | Molybdenum antimony anticolorimetric method |

Table S2 Results of principal coordinate analysis on the 11 measured community functional traits

| Axis | Eigenvalue | Variation explained by individual axis (%) | Cumulative (%) |
| --- | --- | --- | --- |
| 1 | 91.179 | 27.453 | 27.453 |
| 2 | 48.920 | 18.363 | 45.816 |
| 3 | 33.890 | 13.817 | 59.633 |
| 4 | 27.956 | 10.787 | 70.420 |


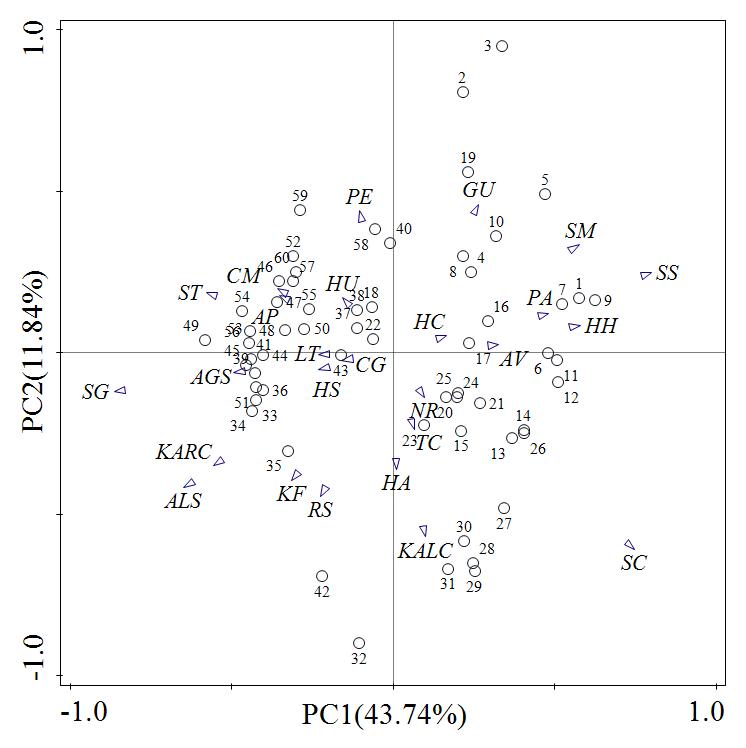


Fig S1 The plant distribution pattern along the transect

Note: 1-60 represents the plot number, the smaller number represent close to the river and the larger number represent far away from the river. *NR*, *PE*, *HH*, *PA*, *GU*, *AV*, *HA*, *SM*, *SS*, *RS*, *TC*, *HS*, *SC*, *ALS*, *HC*, *KF*, *KALC*, *SG*, *KARC*, *CG*, *CM*, *ST*, *AP*, *AGS*, *HU*, *LT* represent *Nitraria roborowskii*, *Populus euphratica*, *Halimodendron halodendron*, *Phragmites australis*, *Glycyrrhiza uralensis*, *Apocynum venetum*, *Haloxylon ammodendron*, *Suaeda microphylla*, *Suaeda salsa*, *Reaumuria soongarica*, *Tamarix chinensis*, *Halocnemum strobilaceum*, *Salsola collina*, *Alhagi sparsifolia*, *Halostachys capsica*, *Kalidium foliatum*, *Kalidium capsicum*, *Suaeda glauca*, *Karelinia caspia*, *Chenopodium glaucum*, *Calligonum mongolicum*, *Seriphidium terrae-albae*, *Aeluropus pungens*, *Agriophyllum squarrosum*, *Horaninovia ulicina* and *Lactuca tatarica*.


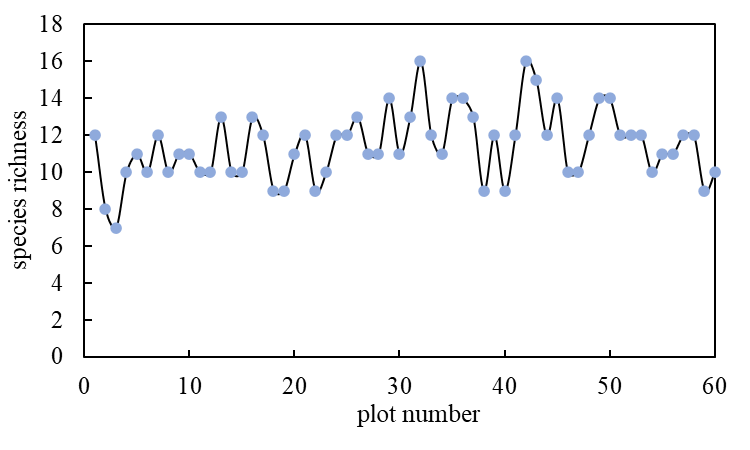


Fig S2 The variation pattern of plant species richness along the transect

Note: 1-60 represents the plot number, the smaller number represent close to the river and the larger number represent far away from the river
